# Genetics, genomics, and breeding of black gram [*Vigna mungo* (L.) Hepper]

**DOI:** 10.3389/fpls.2023.1273363

**Published:** 2024-01-15

**Authors:** Ramakrishnan M. Nair, Sunil Chaudhari, Nagamallika Devi, Aparna Shivanna, Abhishek Gowda, Venkata N. Boddepalli, Hansaraj Pradhan, Roland Schafleitner, Souframanien Jegadeesan, Prakit Somta

**Affiliations:** ^1^ World Vegetable Center, South Asia, Patancheru, India; ^2^ World Vegetable Center, Shanhua, Tainan, Taiwan; ^3^ Bhabha Atomic Research Centre (BARC), Mumbai, India; ^4^ Kasetsart University, Nakhon Pathom, Thailand

**Keywords:** *Vigna mungo*, black gram, Urdbean, breeding, genetics, genomics

## Abstract

Black gram [*Vigna mungo* (L.) Hepper] is a highly nutritious grain legume crop, mainly grown in South and Southeast Asia, with the largest area in India, where the crop is challenged by several biotic and abiotic stresses leading to significant yield losses. Improving genetic gains to increase on-farm yields is the primary goal of black gram breeding programs. This could be achieved by developing varieties resistant to major diseases like mungbean yellow mosaic disease, urdbean leaf crinkle virus, *Cercospora* leaf spot, anthracnose, powdery mildew, and insect pests such as whitefly, cowpea aphids, thrips, stem flies, and bruchids. Along with increasing on-farm yields, incorporating market-preferred traits ensures the adoption of improved varieties. Black gram breeding programs rely upon a limited number of parental lines, leading to a narrow genetic base of the developed varieties. For accelerating genetic gain, there is an urgent need to include more diverse genetic material for improving traits for better adaptability and stress resistance in breeding populations. The present review summarizes the importance of black gram, the major biotic and abiotic stresses, available genetic and genomic resources, major traits for potential crop improvement, their inheritance, and the breeding approaches being used in black gram for the development of new varieties.

## 1 Introduction

Black gram [*Vigna mungo* (L.) Hepper; 2n = 22] is a highly nutritious grain legume crop mainly grown in South and Southeast Asian countries including Afghanistan, Bangladesh, India, Myanmar, Pakistan, Sri Lanka, Thailand, and Vietnam (Kaewwongwal et al., 2015). The crop is also grown on a smaller scale in some African countries such as Kenya, Uganda, and Tanzania and in South American countries such as Argentina and Brazil. Black gram is also locally known as Urdbean, Urid, Mash, and Biri in India; as Mashkalai in Bangladesh; as Maas in Nepal; as Matpe in Myanmar; as Thua Khiao Piu Dam and Thua Khaek in Thailand; and as đậu muồng ăn in Vietnam. It was domesticated in India from its wild progenitor, *V. mungo* var. *silvestris* (Chandel et al., 1984) approximately 3,500 to 4,500 years ago (Fuller and Harvey, 2006). From India, black gram spread to the west coast peninsular of Thailand (Southeast Asia) approximately 2,300 years ago (Castillo, 2013). It plays an important role in vegetarian diets in South Asia due to its high nutritive value. Mature dry seeds of black gram possess approximately 24%–26% protein, 60% carbohydrates, 1.3% fats, phosphorus (345 mg/100 g), potassium, iron (8.7 mg/100 g), and calcium (185 mg/100 g) along with several essential amino acids (arginine, phenylalanine, leucine, lysine, valine, and isoleucine, etc.), vitamins such as vitamin B3 (niacin; 2 mg/100 g), vitamin A (23 IU/100 g), vitamin B1 (thiamine; 0.42 mg/100 g), and vitamin B2 (riboflavin; 0.37 mg/100 g) (USDA National Nutrient Database, 2018). It is widely consumed as dry whole grain or split grain known as *daal* and as unfermented and fermented flour (Khan et al., 2021). Popular Indian dishes like *idli*, *dosa*, and *vada* are prepared using black gram flour. It is also used as a major ingredient in several food items such as cakes, biscuits, snacks, and cookies. Its seed may be used in the food industry as functional food and nutraceutical as well as in the cosmetic and pharmaceutical industries (Pandey, 2019; Khan et al., 2021). In Thailand and Japan, black gram sprouts are preferred to mungbean sprouts because of their longer shelf life (Kaewwongwal et al., 2015).

The crop is a potential component of various cropping systems, especially in rice and wheat fallows owing to its short life cycle (70–90 days), capacity to fix atmospheric nitrogen, and relative drought tolerance. Black gram is generally intercropped with maize, sorghum, cotton, millets, and pigeonpea or rotated with cereal crops such as rice to increase soil fertility, minimize pest and disease incidence, and enhance dry matter yield of main crops (Yadav et al., 2006). India is the world’s largest producer of black gram, contributing 70% of the global production, followed by Myanmar and Pakistan. India produces approximately 2.7 million tonnes from an approximately 4.4 m ha area with an average yield of 598 kg/ha (Directorate of Economics and Statistics, 2021). Approximately 60% of the crop area is cultivated during the *Kharif* season; however, the *rabi* season cultivation is increasing due to the adoption of early maturing (75–80 days) varieties in rice fallows. Black gram contributes approximately 10% of the total pulse production in India with more than 90% of its production coming from 10 states, *viz*., Maharashtra, Karnataka, Madhya Pradesh, Gujarat, Uttar Pradesh, Jharkhand, Telangana, Odisha, Andhra Pradesh, and Tamil Nadu (Directorate of Economics and Statistics, 2021).

In Myanmar and Thailand, black gram has been largely cultivated as an export crop for Indian and Japanese markets since the late 1980s (Kaewwongwal et al., 2015). The crop is grown on approximately 1.1 m ha with a total production of approximately 1.58 million tonnes in lower Myanmar, especially in Bago (44%) and Ayeyarwady regions (41%) (Fujita and Okamoto, 2006; Dasgupta and Roy, 2015). In Australia and the USA, it is also grown as a fodder crop (Jansen, 2006). Due to the slow rate of production growth, India has become increasingly dependent on imports from some of the other black gram-producing countries such as Myanmar, Kenya, Mozambique, Australia, and Tanzania to satisfy domestic black gram demand (Commodity Profile for Pulses, 2019). Low and stagnant productivity (450–800 kg/ha) is one of the major stumbling blocks for vertical as well as horizontal expansion of the crop. Limited active breeding programs across major growing countries focusing on black gram improvement, lack of access to genetic and genomic resources, and poor seed systems are some of the major reasons behind low productivity levels (Kaewwongwal et al., 2015). The manuscript reviews the major production constraints, nature of inheritance of different desirable traits, and genetic and genomic resources available to enhance the genetic gains for target traits in black gram breeding.

## 2 Black gram production constraints

The gap between potential and realized yield in pulses including black gram is wide and could be attributed to several biotic and abiotic stresses and the cultivation of the crop under poor crop management conditions (Rana et al., 2016). These biotic and abiotic stresses vary across the production regions depending on the cropping system and prevailing weather conditions. Understanding these production constraints is important for breeding programs to design a sound strategy to improve the yield levels of black gram.

### 2.1 Biotic stresses

The prevailing mono-cropping and intensive farming systems created the problem of several pests and diseases, and their intensive management through chemical measures has resulted in the development of resistance in pathogens against chemicals (Saxena et al., 2018). In India, biotic stresses including pests and diseases are reported to cause yield losses of up to 70% in black gram (Sharma et al., 2011), of which nearly 30% is due to insects (Justin et al., 2015). However, the avoidable losses due to pest incidence varied from 15.62% to 30.96% with an average of 24.03% in black gram production (Duraimurugan and Tyagi, 2014). It indicates that the loss due to the biotic stresses can be minimized through adopting resistant cultivars and integrated pest and disease management practices.

#### 2.1.1 Insect pests

Approximately 198 insect species are reported to feed on pulse crops around the world, of which 115 are reported in India (Kooner et al., 2006). Out of these 115 species, 45 insect species have been reported on mungbean and black gram (Saxena et al., 2018), approximately 25 of them were major insect pests of black gram during the *Kharif* season, and 17 were reported during the spring season (Kooner et al., 2006; Duraimurugan and Tyagi, 2014). The insect species composition and their infestation vary across geography, seasons, plant phenology, and the prevailing environmental conditions such as temperature, humidity, and rainfall. The pest spectrum of black gram (**Table 1**) could be divided based on their feeding habit and plant parts as a) defoliators, *viz.*, Bihar hairy caterpillar (*Spilosoma obliqua* Walker), red hairy caterpillar (*Amsacta moorei* Butler), tobacco cutworm (*Spodoptera litura*Fabricius), and blue butterfly (*Lampides boeticus* (Linnaeus)); b) sucking pests, *viz.*, leafhopper (*Empoasca kerri* Pruthi), whitefly (*Bemisia tabaci* Gennadius), cowpea aphids (*Aphis craccivora* Koch), and thrips (*Megalurothrip distalis* Kany and *Caliothrips indicus* Bagnall); c) internal feeders, *viz.*, stem fly (*Ophiomyia phaseoli* Tryon) and Galerucid Beetles (*Madurasia obscurella* Jacoby); d) pod borers/pod sucking bugs, *viz.*, plant bugs (*Riptortus pedestris* (Fabricius), *Nezara viridula* (Linnaeus), and *Clavigralla gibbosa* Spinola), spotted pod borer (*Maruca vitrata* Fabricius), field bean pod borer (*Adisura atkinsoni* Moore), and bruchids (*Callosobruchus chinesis* Linnaeus and *Callosobruchus maculatus* Fabricius) (Sharma et al., 2011; Yadav et al., 2015; Saxena et al., 2018).

**Table 1:** Summary of major insect pest complex in black gram.

Insect pest	Vulnerable plant stage	Plant parts affected	Preferred season of incidence	Estimated yield losses (%)	Reference
a) Defoliators					
Bihar hairy caterpillar (*Spilosoma obliqua*)	All the stages	Larvae feed on leaf and young shoot	Rainy and Post-rainy	30%–35%	Berani et al., 2018
				70%–80%	Prasad et al., 2005
Tobacco cutworm (*Spodoptera litura*)	All the stages	Young larvae feed on leaves; older larvae feed on flowers and pods	Rainy	80%	Kitturmath, 2008
b) Sucking pests					
Whitefly (*Bemisia tabaci*)	All the stages	Adults and nymphs suck sap from underside of leaves	Rainy	7–71	Mahalakshmi et al., 2015
Cowpea aphid (*Aphis craccivora*)	All the stages	Adults and nymphs congregate on leaves, flowers, young pods, and stems	Rainy		
Thrips (*Megalurothrips distalis*)	Seedling and flowering stages	Adults and nymphs suck sap from growing shoots, young leaves, and flowers	Spring	23–50	Sharma et al., 2011
c) Internal feeders					
Stem fly (*Ophiomyia phaseoli*)	Seedling stage	Maggots penetrate trifoliate leaf and move to succulent stem	Rainy/post-rainy	3–62	Lal, 1985
Galerucid Beetles (*Madurasia obscurella*)	Young seedling	Adults feed on leaf and the root nodules	Rabi	60	Saxena et al., 2018
d) Pod borers/pod sucking bugs					
Pod sucking bugs (*Riptortus pedestris*, *Nezara viridula*, *Clavigralla gibbosa*)	Green pod stage	Adults and nymphs suck sap from leaves, young pods, and flowers	Post-rainy	5–18	Saxena et al., 2018
Pod borer complex					
Spotted pod borer (*Maruca vitrata*)	Flowering to pod maturity	Larvae feed on flowers and maturing pods	Rainy	15–20	Saxena et al., 2018
				24%	Ramu et al., 2018
Field bean pod borer (*Adisura atkinsoni*)	Flowering to pod maturity	Larvae feed on flowers and maturing pods	Rainy		
Pod borer *(Helicoverpa armigera)*	All stages	Young larvae feed on leaf; older larvae feed on flowers and pods	Rainy		
Bruchids (*Callosobruchus* spp)	Post-harvest storage	Seeds	All seasons	20%–100%	Souframanien et al., 2011

Sucking pests are the major group of insects that cause yield reduction in three ways: a) direct feeding/sucking on the plant parts such as leaves, flowers, young shoots, and pods could lead to poor growth and development of the plant; b) by acting as a vector for viral diseases; and c) by hampering the photosynthesis, causing poor nutrient assimilation. The flower thrips feed on the floral parts, and the flower shed before opening; thus, plants attain a bushy growth bearing few pods with shriveled seeds (Sharma et al., 2011; Srinivasan, 2014). Some of the sucking pests such as whitefly is a vector for viral diseases such as mungbean yellow mosaic disease (MYMD) and *Urdbean Leaf Crinkle Virus* (ULCV) and are responsible for direct as well as indirect yield losses. Dropping of young pods was reported under the severe incidence of cowpea aphid and pod bugs (Sharma et al., 2011). Cowpea aphids excrete honeydew while feeding on plants, which leads to the development of sooty molds on plants, leading to poor photosynthesis, nutrient assimilation, and yield levels (Annan et al., 1994).

The sucking pests such as whitefly and aphids, pod feeders, and pod bugs were reported to cause more damage during the rainy season, whereas thrip incidence was reported to be severe during the spring season, which could be due to higher temperatures during the spring season (Duraimurugan and Tyagi, 2014; Yadav and Patel, 2015). The stem fly is another major pest in some of the black gram growing states of India such as Punjab (Saxena et al., 2018) and Odisha and Bihar (Yadav and Patel, 2015) and is recently reported as an emerging pest in Andhra Pradesh (Manjula et al., 2019). The maggots of stem fly mine the leaves or bore into the leaf petiole and tender stem, resulting in withering, drooping, and death of the plant, leading to yield losses of 20%–60% to the complete crop failure if not managed during the seedling stage (Lal, 1985; Sharma et al., 2011; Saxena et al., 2018; Manjula et al., 2019).

The young larvae of *Helicoverpa armigera* damage the crop by defoliation, while the older ones prefer to feed on buds, flowers, and pods. The adults of the spotted pod borer arrive on the plants during the flowering stage to lay eggs on the flowers. Emerging larvae web the leaves and inflorescence and devour the flowers, flower buds, and pods. The larval feeding holes on pods act as entry holes for the moisture leading to the discoloration of seeds, thus reducing market quality (Lal, 1985; Sharma et al., 2011; Srinivasan, 2014; Saxena et al., 2018). Bruchids are important storage pests of black gram, which could cause 100% yield loss if not managed while storing the grains. It reduces seed weight, germination, nutritional quality, and market value, thereby rendering it unfit for human consumption, agricultural, and commercial uses (Duan et al., 2014). The study on the effect of bruchid infestation on antinutritional factors in legumes reported increases in trypsin inhibitor activity by 25%, saponin by 16%, and phytic acids by 46% (Modgil and Mehta, 1997; Baroowa and Gogoi, 2012). The seed moisture above 10% increases the susceptibility of grain to bruchids (Sharma et al., 2011). The important insect pests of black gram and respective information on the economic damage, season, and preferred plant parts for feeding are summarized in **Table 1**.

#### 2.1.2 Diseases

Among the diseases, MYMD caused by *Mungbean Yellow Mosaic Virus* (MYMV) and *Mungbean Yellow Mosaic India Virus* (MYMIV) is one of the major production constraints, responsible for more than 50% yield reductions in black gram and other *Vigna* species in South and Southeast Asia. In addition to black gram, MYMD is also responsible for the reduction in yields of other leguminous crops (Ramesh et al., 2017; Dikshit et al., 2020). The MYMV belongs to the Gemini group of viruses mainly transmitted by the whitefly. Initially, yellow spots appear on the leaves, and eventually, these yellow spots coalesce, turning the complete vegetation into a yellow color. The leaf yellowing reduces photosynthesis and nutrient assimilation, leading to poor flowering and pod development (Srivastava and Prajapati, 2012). Considering the devastating incidence of MYMD across production environments, it has become one of the must-have traits for breeding programs along with other economically important traits. Urdbean leaf crinkle disease (ULCD) is another viral disease considered to be very severe in black gram compared to other *Vigna* species (Kadian, 1980; Bashir et al., 2005). It is responsible for the reduction of seed yield from 35% to 81%, depending upon the time of infection, variety under cultivation, and growing season (Bashir et al., 1991; Reddy et al., 2005; Ashfaq et al., 2007; Gautam et al., 2016). However, some studies reported approximately 76%–100% yield reductions due to ULCD (Singh, 1980). The disease is broadly distributed across different states in India (William et al., 1968) and Pakistan (Bashir and Zubair, 1985; Ilyas et al., 1992). ULCV belongs to the Tospovirus group and is reported to be transmitted by several insects, *viz.*, aphids (Dhingra, 1975), whiteflies, and leafhoppers (Gautam et al., 2016). The viral disease causes crinkling, curling, and puckering of leaves; the infected plants become stunted with deformed floral organs (Reddy et al., 2005). The leaf crinkle disease-susceptible plant produces smaller seeds, leading to poor yields. Disease incidence during seedling to vegetative stage caused more yield losses compared to incidence during reproductive stages (Reddy et al., 2005).

*Cercospora* leaf spot (CLS) caused by *Cercospora canescens* is another important disease that mostly affects rainy season crops owing to a reduction in yields by 25% when leaf defoliation reaches 75% (Dhar and Chaudhary, 2001; Gupta et al., 2020). This pathogen forms spots on the leaves with a brown to grayish center and reddish-brown border, which subsequently spread to petioles, pods, and stems. The disease leads to severe defoliation under favorable humid conditions (Raguchander et al., 2005; Dubey and Singh, 2010; Singh, 2010).

Powdery mildew caused by *Podosphaera xanthii* became another production constraint and priority for black gram breeding programs, especially in the agro-ecologies where the crop is grown in rice fallows. The symptom initiates as feeble dark spots on the leaf, which develop into small white powdery spots, coalescing to form a white powdery coating on all the aerial plant parts such as leaves, stems, and pods. Subsequently, the color of the powdery mass turns dirty white and covers the entire leaf surface, leading to reduced photosynthesis and induced early maturity and subsequent yield reduction. Unlike CLS disease, it is severe under cooler conditions (Sharma et al., 2011). In addition to these, focus also needs to be given to some of the emerging diseases such as anthracnose, *Macrophomina* blight, and bacterial leaf spot. *Macrophomina* blight is caused by the *Macrophomina phaseolina* responsible for dry root rot, collar rot, seedling blight, stem rot, leaf blight, and pod and stem blight (Vidhyasekaran and Arjunan, 1978; Sharma et al., 2011). The seedling rot during germination reduces the crop establishment. The infected plants form localized dark brown patches at ground level, which later encircle the stem. The sclerotial bodies appear on the outer tissue of the stem and root (Basandrai et al., 2016).

The anthracnose disease is mainly caused by *Colletotrichum truncatum* (Basandrai et al., 2016; Saxena et al., 2018) and seldom by *Colletotrichum capsici* (Mishra et al., 2011). The fungal pathogen forms characteristic circular brown sunken spots with dark centers and bright red-orange margins produced on the leaves and pods. Under severe disease situations, affected plants fall off. The pathogen is reported to be transmitted to the next generation through seeds and crop residue (Mishra et al., 2011). The summary of major diseases, their estimated yield losses, and favorable weather conditions for disease development are presented in **Table 2**.

**Table 2:** Summary of major black gram diseases, their causal agents, reported yield losses, and optimal weather conditions for disease development.

Disease	Causal agent	Optimal weather conditions	% Yield loss	Reference
Leaf Crinkle Disease	*Urdbean Leaf Crinkle Virus*	Cooler weather; temperature of 15°C–20°C; RH >90%	2%–95%	Kadian, 1980 Negi and Vishunavat, 2006
			81%	Bashir et al., 1991
Yellow Mosaic Disease	*Mungbean Yellow Mosaic Virus*	Humid weather	15%–25%	Dubey and Singh, 2010
Anthracnose	*Colletotrichum truncatum*	Intermittent rains; temperature of 17°C–24°C; RH 100%	39%–65%	Chatak and Banyal, 2020
Powdery Mildew	*Erysiphae polygoni*	Humid weather; the temperature of 27.2°C–30.3°C; RH 90%	9%–45%	Pandey et al., 2009
Root rot/seedling blight	*Macrophomina phaseolina*	Warmer condition >30°C and water stress	34%–54%	Singh et al., 1998
*Cercospora* Leaf spot	*Cercospora canescens*	Humid weather	20%–36%	Dubey and Singh, 2010

### 2.2 Abiotic stresses

Among the abiotic stresses, drought, heat, waterlogging, and salinity stresses along with photo- and thermo-sensitivity are some of the major abiotic stresses responsible for severe yield losses in legumes across the production environment (Sultana et al., 2014; Nadeem et al., 2019). These stresses pose physiological changes to the plant and adversely affect the yield and nutritional quality of legume crops (Scheelbeek et al., 2018; Zonneveld et al., 2020). Black gram cultivation is performed year-round during rainy, post-rainy, and spring seasons across different agro-ecologies and cropping systems in India and other countries. In humid regions of North-East and coastal parts of India, black gram is grown soon after the monsoon rice crop to utilize the residual soil moisture, whereas it is grown as a rainfed crop in several parts of North India (Punjab, Haryana, Western Uttar Pradesh, and Bihar) (Singh et al., 2009). The water deficit stress is often experienced by the crop in many regions, especially during the rainfed and post-rainy season cultivation without supportive irrigation. Approximately 20%–30% of yield losses could be due to water deficit stress, which can be higher if the stress coincides with the flowering and pod development stage (Baroowa and Gogoi, 2012; Sai and Chidambaranathan, 2019; Kumar et al., 2020). It also reduces the efficiency of physiological processes such as the rate of photosynthesis and transpiration, and stomatal conductance (Durga et al., 2003; Baroowa and Gogoi, 2015). Moisture stress disturbs turgor pressure in the plant cells and affects cell enlargement, photosynthetic pigments, and membrane stability, resulting in poor plant growth. In addition to these, it also affects the stress response, defense systems of plants against pathogens, and several gene expressions and signaling pathways (Baroowa and Gogoi, 2015). The moisture deficit stress-tolerant varieties, *viz.*, VBN4 and K1, showed a fivefold increase in production of abscisic acid (ABA) and lipid peroxidase activity compared to susceptible varieties (Sai and Chidambaranathan, 2019).

In several studies, the stress-tolerant black gram genotypes exhibited higher quantities of chlorophyll and increased leaf area index (Pratap and Sharma, 2010; Kumar et al., 2020), plant height, stress tolerance index, dry matter stress tolerance index, and assimilation rate (Baroowa and Gogoi, 2015; Yohan et al., 2018) compared to the reduction in these growth parameters in stress-susceptible genotypes (Gurumurthy et al., 2019; Shahi et al., 2019). Soil salinity reduces the chlorophyll content and affects leaf turgidity while increasing the production of proline (Ashraf, 1989).

The black gram is sensitive to low as well as higher temperature conditions. The high-temperature stress is responsible for the reduction of 38% to 82% of seed yield in black gram (Anitha et al., 2015). The pollen germination was significantly affected during higher temperatures. The plant traits such as leaf morphology (wax/pubescence), seed hardiness, pollen viability, and germination and receptivity of stigma were associated with temperature tolerance (Ganeshan et al., 2012; Choudhary et al., 2014). The higher temperature-tolerant (>38°C ± 2°C) genotype produced seed surface with shiny luster, thin and reduced cotyledon fissures, and bold and well-structured starch granules (Partheeban and Vijayaraghavan, 2020). Considering the changing climate scenario, breeding for climate-smart varieties that offer tolerance to prevailing biotic and abiotic stresses will ensure uninterrupted growth in genetic gains and supply of increasing future food demand.

## 3 Genetics

Enhancement of yield under various biotic and abiotic stresses requires the analysis of trait diversity found in germplasm collections and the study of inheritance patterns of important morphological and agronomic traits. There are some studies on genetic diversity in black gram for agronomic and morphological traits (Ghafoor et al., 2001; Shafique et al., 2011; Choudhary et al., 2018; Patidar et al., 2018), seed storage protein (Ghafoor and Ahmad, 2005), grain yield (Kumar et al., 2002; Tomar et al., 2003), and resistance against biotic stresses (Vishalakshi et al., 2017). However, studies on the genetics and inheritance of qualitative and quantitative morphological and agronomic traits, the cytogenetics, and the combining ability among genotypes for different target traits are limited in black gram (Kumar et al., 2000) as compared to other *Vigna* species such as cowpea and mungbean. Based on the available studies, the inheritance pattern of different morphological, biotic, and abiotic stress-related traits is discussed below.

### 3.1 Genetics of morphological traits

Black gram shows a spreading plant type; however, the modern cultivars are developed with erect and semi-erect plant types. The erect plant type is not completely dominant over the spreading type (Sen and Jana, 1964). It has large trifoliate leaves that are either ovate or lanceolate in shape. Ovate leaf shape is dominant over lanceolate and is mainly controlled by a single dominant gene (Verma, 1971). However, the hastate shape is reported to be dominant over the ovate and probably controlled by duplicate dominant genes (Singh and Singh, 1971) (**Table 3**). A fused leaf (cotton leaf type) variant was reported to be recessive to the ovate leaf shape and is controlled by a single recessive gene (Muralidharan et al., 1990). Two types of pod orientations, i.e., main stem bearing and sympodial bearing types, are found in black gram. Main stem pod bearing is controlled by a single incomplete dominant gene. Recently, the above canopy pod-bearing genotypes were also developed in black gram (Gupta et al., 2020). Three different pod colors are reported, *viz.*, black, brown, and straw color, of which black pod color is very prominent and dominant over the straw and brown pod color (Sen and Jana, 1964; Verma, 1971; Arshad et al., 2005). The inheritance of protruded stigma and crumpled petals in naturally occurring mutants reported the involvement of a single recessive gene with a pleiotropic effect (Kumar et al., 2012). The brown seed coat color is recessive to the green seed coat color with qualitative inheritance (Sen and Jana, 1964). However, in contrast to this, brown seed coat color was reported to be dominant over green seed color by Arshad et al. (2005). Shiny seed surface is dominant over dull seed surface in black gram (Sen and Jana, 1964).

**Table 3:** Nature of inheritance of some of the major traits of black gram.

Characters	Number of genes and nature of inheritance	References
Growth habit	Erect plant type is not completely dominant over spreading type	Sen and Jana, 1964
Terminal leaflet type	Single dominant gene Ovate leaf shape is dominant over lanceolate	Verma, 1971
	Controlled by duplicate gene Hastate leaf shape is dominant over ovate	Singh and Singh, 1971
	Multifoliate leaf is controlled by a single recessive gene	Rao et al., 1989
	Controlled by a single recessive gene Fused leaf variant is recessive to ovate leaf shape	Muralidharan et al., 1990
Pod pubescence	Controlled by a single dominant gene Hairy pods are dominant over non-hairy pods	Pathak, 1961; Sirohi and Singh, 1998; Arshad et al., 2005
Pod bearing	Main stem bearing was controlled by single gene with incomplete dominance	Rao, 1999
Pod color	Single dominant gene Black pod color is dominant over straw and brown pod color	Sen and Jana, 1964 Verma, 1971 Arshad et al., 2005
Flower shape	Monogenic recessive inheritance Malformed flower recessive to normal flower	Jana, 1962
Protruded stigma	Single recessive gene with pleiotropic effects	Kumar et al., 2012
Seed coat color and seed luster	Controlled by a single gene Brown seed coat color recessive to green seed coat color	Sen and Jana, 1964
	Brown seed coat color dominant over green seed coat color	Arshad et al., 2005
	Shiny seed luster dominant over dull seed luster	Sen and Jana, 1964
Mungbean yellow mosaic disease	Monogenic dominant	Dahiya et al., 1977; Kaushal and Singh, 1988a; Gupta et al., 2005; Gupta et al., 2013a
	Monogenic and digenic recessive	Singh, 1981; Dwivedi and Singh, 1985; Verma and Singh, 1986; Singh et al., 1987; Pal et al., 1991; Rambabu et al., 2018
	Duplicate dominant gene interaction	Thamodhran et al., 2016
	Complementary or duplicate recessive epistatic gene interaction	Thamodhran et al., 2016
	Tri-genically controlled with inhibitory gene action	Vadivel et al., 2021
	Complementary gene action with two genes	Vadivel et al., 2021
Powdery Mildew	Dominant monogenic inheritance	Srivastava et al., 2013; Santosh, 2016
	Single recessive gene	Kaushal and Singh, 1989
*Cercospora* Leaf Spot	Dominant gene	Kaushal and Singh, 1991
Leaf spots (*Colletotrichum truncatum*)	Single dominant gene	Kaushal and Singh, 1988b
Urdbean leaf crinkle disease	Digenic inhibitory gene action (13 resistance:3 susceptible)	Sathya et al., 2022
	Trigeneic inhibitory gene action (49R:15S)	
Bruchid infestation (*C. maculatus*)	Dominant duplicate gene	Dongre et al., 1996

### 3.2 Genetics of resistance to biotic stresses

There are contrasting reports on the inheritance of resistance that include monogenic dominant (Dahiya et al., 1977; Kaushal and Singh, 1988a; Gupta et al., 2005; Gupta et al., 2013a), as well as the monogenic or digenic recessive nature of resistance to MYMD (Singh, 1981; Dwivedi and Singh, 1985; Verma and Singh, 1986; Singh et al., 1987; Pal et al., 1991; Rambabu et al., 2018) available in black gram. The duplicate and recessive epistatic gene interaction (Thamodhran et al., 2016) along with tri-genic control with inhibitory gene action (Vadivel et al., 2021) was also reported for resistance to MYMD. The chi-square goodness-of-fit test on an F_2_ population revealed inhibitory gene action with two genes controlling the expression of resistance to MYMD (Subramaniyan et al., 2022). This suggests that the genetics of resistance to MYMD is complex, which could be due to the presence of different strains of the causal virus and their variable pathogenicity. Genetic analysis of resistance to PM reported it to be governed by a single recessive gene (Kaushal and Singh, 1989); however, a recent report indicates that the resistance is controlled by a single dominant gene without maternal effects (Santosh, 2016). The inheritance of resistance to CLS (Kaushal and Singh, 1991) and anthracnose is controlled by a single dominant gene (Kaushal and Singh, 1988b). Two duplicate dominant genes control resistance to bruchid (*C. maculatus*) infestation in black gram (Dongre et al., 1996; Souframanien and Gopalakrishna, 2007). However, resistance is also reported to be controlled by the monogenic dominant gene (Tickoo et al., 2006) (**Table 3**).

## 4 Genetic resources

The loss of genetic diversity during domestication and subsequent diversification of crops resulted in a decrease in the genetic diversity and useful genes/traits that were not selected during domestication. The limited information on the characterization and evaluation of germplasm accessions led to the use of fewer parents by the breeding programs, resulting in a narrow genetic base in most of the *Vigna* species. Screening of diverse germplasm accessions and crop wild relatives can provide valuable genetic resources to source new traits for breeding programs (Ganguly and Bhat, 2012).

### 4.1 Black gram germplasm

As black gram originated in India, the largest number of its globally available genetic resources (3,146 accessions) are collected and conserved by the National Bureau of Plant Genetic Resources (NBPGR), New Delhi, India (Singh D et al., 2016). The ICAR-Indian Institute of Pulses Research (ICAR-IIPR), Kanpur, India, has the mandate of pulse crop improvement including black gram and also holds an active collection of approximately 829 accessions (Arora, 1988; Gupta et al., 2001). The largest black gram collection outside of India is held by the World Vegetable Center, Taiwan, comprising 884 black gram accessions that originated from different parts of the world (Jacob et al., 2015). Some other gene banks are holding black gram, *viz.*, Plant Genetic Resources Centre (PGRC), Bangladesh Agricultural Research Institute (BARI), Bangladesh (339 accessions); Plant Genetic Resources Conservation Unit, University of Georgia, USA (304 accessions); N.I. Vavilov All-Russian Institute of Plant Genetic Resources, Russia (220 accessions); and Research Center of Genetic Resources, National Agriculture and Food Research Organization (NARO), Japan (220 accessions). Australian Grains Genebank, Australia (102 accessions) and International Center for Tropical Agriculture, Colombia (93 accessions) also hold black gram accessions (**Table 4**). NARO has the largest number of accessions (14) of wild black gram. However, many germplasms conserved in several gene banks are duplicates of Indian origin. There is considerable genetic diversity available among the Indian black gram accessions (Singh et al., 1991; Singh and Shukla, 1994; Nautiyal and Shukla, 1999; Kaewwongwal et al., 2015); however, its exploitation was hindered by limited access to information about the germplasm such as characterization data and classification efforts (Gupta et al., 2001).

**Table 4:** Availability of Black gram germplasm accessions across the world.

S. no.	Name of institute/gene bank	Country	# Accessions	Reference
1	Australian Grains Genebank	Australia	102	https://ausgenebank.agriculture.vic.gov.au
2	Bangladesh Agricultural Research Institute (BARI)	Bangladesh	339	Bhisht and Singh, 2013
3	Botanic Garden Meise	Belgium	12	www.genesys-pgr.org
4	Embrapa Genetic Resources and Biotechnology	Brazil	9	(www.cenargen.embrapa.br)
5	Institute for Plant Genetic Resources	Bulgaria	7	www.genesys-pgr.org
6	Centro de Investigación La Selva (CoRPOICA)	Colombia	108	Bhisht and Singh, 2013
7	Alliance of Biodiversity International and (CIAT)	Colombia	93	www.genesys-pgr.org
8	Institute for Agrobotany	Hungary	17	www.genesys-pgr.org
9	National Bureau of Plant Genetic Resources (NBPGR)	India	3,146	Singh et al., 2016a
10	ICAR-Indian Institute of Pulses Research	India	829	Gupta et al., 2001
11	Genetic Resources Research Institute	Kenya	13	www.genesys-pgr.org
12	NARC, Lalitpur Kathmandu	Nepal	83	Bhisht and Singh, 2013
13	International Institute of Tropical Agriculture	Nigeria	11	https://www.genesys-pgr.org/10.18730
14	Pakistan Agricultural Research Council, PGRI/NARC	Pakistan	693	Bhisht and Singh, 2013
15	National Institute of Agri Biotechnology and Genetic Resources, National Agricultural Research Centre, Pakistan Agricultural Research Council (PARC)	Pakistan	134	Kaewwongwal et al., 2015
16	N.I. Vavilov All-Russian Institute of Plant Genetic Resources	Russia	220	https://www.vir.nw.ru
17	World Vegetable Center	Taiwan	884	http://seed.worldveg.org/search/passport
18	National Agriculture and Food Research Organization	Japan	210	https://www.gene.affrc.go.jp
19	Plant Genetic Resources Conservation Unit, Southern Regional Plant Introduction Station, University of Georgia, USDA-ARS	USA	304	www.ars-grin.gov
20	USDA, Agricultural Research Service, National Plant Germplasm System. 2021. Germplasm Resources Information Network (GRIN Taxonomy). National Germplasm Resources Laboratory, Beltsville, Maryland	USA	148	USDA, Agricultural Research Service, National Plant Germplasm System 2021 https://npgsweb.ars-grin.gov/gringlobal/taxon/taxonomysearchcwr

Molecular genetic diversity analysis of 520 cultivated and 14 wild accessions from various countries using 22 simple sequence repeat (SSR) markers revealed that the diversity of germplasm from different regions was comparable, albeit the germplasm from South Asia showed the greatest gene diversity (Kaewwongwal et al., 2015). The study also revealed that the level of gene diversity in black gram is close to that in mungbean and rice bean, but lower than azuki bean.

### 4.2 Tapping useful genes from wild relatives

Black gram is classified into two sub-taxa, i.e., i) *V. mungo* var. *mungo* characterized by black large seeds and early maturity and the wild type ii) *V. mungo* var. *silvestris* Lukoki, Maréchal & Otoul, with denser inflorescences, hairier and climbing nature, and small seeds with prominent raised aril (Lukoki et al., 1980; Tomooka et al., 2000). *V. mungo* var. *silvestris* is considered to be the progenitor of cultivated black gram (Lukoki et al., 1980) and was successfully used in breeding programs for the genetic improvement of several traits (Jansen, 2006). Black gram is classified into the subgenus *Ceratotropis* (also known as Asian Vigna). Species in this subgenus are highly diverse and distributed widely in Asia (Tomooka et al., 2000), and thus, they adapt well to various environments and can be useful gene sources for the genetic improvement of black gram. Some of the important wild species of *Vigna*, namely, *Vigna bourneae*, *Vigna capensis*, *Vigna dalzelliana*, *Vigna grandis*, *Vigna hainiana*, *Vigna minima*, *V. mungo* var. *sylvestris*, *Vigna radiata* var. *sublobata* (wild mungbean), and *Vigna vexillata*, were collected from Western and Eastern Ghats, Northwestern Plains, Central Plateau region, and Northern Himalayas in India (Dana, 1998). Several accessions of wild *Vigna* species, i.e., *Vigna aconitifolia*, *V. dalzelliana*, *V. hainiana*, *Vigna khandalensis*, *V. mungo* var. *silvestris*, *V. radiata* var. *sublobata*, and *Vigna trilobata*, were collected from diversity rich areas of Gujarat, Rajasthan, Maharashtra, Madhya Pradesh, Bihar, Odisha, and West Bengal during 1974 and 1994 by NBPGR, New Delhi (Dana, 1998). However, there is no information on the cross-compatibility between a black gram and all of these wild *Vigna* species, except wild black gram and wild mungbean.

The cross-compatibility study of *V. mungo* with other species revealed 37.5% compatibility between *V. radiata* (♀) and *V. mungo* (♂), whereas it was 11.6% between *V. mungo* (♀) and *Vigna umbellata* (♂) (Bhanu, 2018). The true interspecific F_1_’s among *V. radiata* (♀, SML 668 and SML 832) and *V. mungo* (♂, Mash 114 and Mash 218) reported 5.5% to 24.1% pod set, 14.29% to 30.56% germination, and 22.59% to 28.36% pollen fertility (Lekhi et al., 2018). Different kinds of pre- and post-fertilization barriers were responsible for complete sterility or low fertility in F_1_ hybrids between these two species (Bhanu, 2018). Rice bean (*V. umbellata*) genotypes RBL 1 and RBL 9 showed high crossability and percent seed set with different cultivars of black gram. Another study successfully crossed *V. mungo* and *V. radiata*, and the recovered hybrids were reciprocally crossed with *Vigna angularis*. The reciprocal three-way interspecific F_1_ hybrids were reported partially fertile (Gupta et al., 2002). The introgression of desirable traits from rice bean (RBL 1) into black gram (Mash 338 and UG 562 and 844) resulted in poor cross-compatibility and plant fertility for initial generations and improved gradually from the F_2_ generation onward (Singh et al., 2013). The yield evaluation of 24 uniform F_7_ progeny bulks exhibited −35.48% to 50.31% yield variation over the check cultivar (‘Mash 338’, a female parent) along with resistance to MYMD, CLS, and bacterial leaf spot diseases. The desirable traits such as a high number of pods per plant, seed weight, and MYMD resistance from rice bean have been successfully introgressed into the black gram.

Varieties Mash 1008 and Mash 118 released in 2004 and 2008, respectively, were developed from an interspecific cross between black gram and mungbean (Singh et al., 2016a). The variety Mash 114 was developed from an interspecific cross between black gram and rice bean and recorded a 39.45% superior yield over the check cultivar ‘Mash 338’ (female parent) across 14 multi-location trials (Singh et al., 2013). Another variety, Vamban 7, resistant to MYMD and powdery mildew was obtained from Vamban 3 × wild black gram (*V. mungo* var. *silvestris*), whereas TU-40, resistant to powdery mildew, was obtained from TU 94-2 × wild black gram (Singh et al., 2016a).

### 4.3 Trait-specific genotypes

The efforts in characterization, evaluation, and screening of black gram germplasm for desirable traits identified several useful trait-specific genotypes for breeding programs. The trait-specific genotypes were identified as potential donors for some of the agronomic traits such as number of pods per plant (KL 1, UH 81-44, and IPU 99-79 with >150 pods), number of seeds per pod (IC 106088, HPU 193, HPU 2, and PLU 257 with >8 seeds per pod), early maturity (IPU96-3, IPU 31-5, PLU 710, L25-7, and STY 2848 <70 days), and MYMD resistance (IC 27026, IC 06088, UL 2, HPU 4, HPU188, STY 2848, UH 80-26, IP 99-127, PLU 62, PLU 158, and PLU227) (Gupta et al., 2001). JP219132 was reported to show extra-large seed size, leaves, and stems (Chaitieng et al., 2006; Naito et al., 2017).

Black gram germplasm identified for biotic stress resistance are compiled in **Table 5**. The genotype LBG-645 was reported as highly resistant to powdery mildew in glass houses and laboratory screening (Priyanka et al., 2018). The genotypes CO-5, IPU 07-3, and Mash 1-1 exhibited moderate resistance to dry root rot compared to the susceptible check VO 2135-B-BL in sick pot assay (Elmerich et al., 2022). Cultivated black gram is known to be immune to *Callosobruchus chinensis* infestation but highly susceptible to *C. maculatus* (Srinives et al., 2007). However, wild progenitor *V. mungo* var. *silvestris* is resistant to *C. maculatus* with the larval antibiosis mechanism of resistance (Dongre et al., 1996; Tomooka et al., 2000). The genotypes that are resistant to spotted pod borer (Krishna et al., 2006) and stem fly were also reported in black gram (Neupane et al., 2021).

**Table 5:** Sources of resistance to major biotic stresses in black gram.

Trait	Level of resistance/tolerance	Identified genotype	Disease score	Reference
MYMD	Resistant	Narendra Urd-1 (NDU-88-1), WBU 108, Mash 338, Birsa Urd-1, Vamban 2, KU 301, IPU-94-1 (Uttara), KU 300, NDU-99-2, KU 96-3	1	Goyal and Khan, 2010
		IC 27026, IC 06088, UL 2, HPU 4, HPU 188, STY 2848, UH 80-26, IP 99-127, PLU 62, PLU 158 and PLU	1	Gupta et al., 2001
		Vamban 7, Vamban 2, Prasad (B 3-8-8), Ujala (OBG-17), Prasad (B 3-8-8) and Ujala (OBG-17), Pant U-31, Pant U-40, Pant U 84, UPU 2	1	Singh et al., 2016a
		TU-94-2, Sarla; VBN 8; PU 31	1	Solanki et al., 2011; Singh et al., 2015; Pandiyan et al., 2018
		TU-94-2, TAU5; Sarla	1	Pawar et al., 2000
		IC 27026, IC 06088, UL 2, HPU 4, HPU188, STY 2848, UH 80-26, IP 99-127, PLU 62, PLU 158, and PLU227	1	Gupta et al., 2001
		IC144901, IC001572, IC011613, IC485638, IC0570265, IC0570262	1	Rana et al., 2016
		Uttara, JU 3, DPU 88-1, DPU 88-31, K 66-10, NDU 88-8, NP 16, NP 19, UG 135, Pant U 19	1	Gaur and Chaturvedi, 2004
		Mash 1-1; TAU-1	1	Solanki et al., 2011
Powdery mildew	Resistant	LBG-645, K-5-572, IC-281978, AKU-10-1, AKU-11-2, LBG-752, LBG-623		Priyanka et al., 2018
		Vamban 7, TU 40	1	Singh et al., 2016a
		WBG 26;	1	Goyal and Khan, 2010
		LBG 645, K-5-572; IC-281978, AKU-10-1, AKU-11-2, LBG-752 LBG-623, LBG-20, IC-436065, KUG-216, BDU-4,	1	Priyanka et al., 2018
Cercospora leaf spot	Tolerant	IC-91567, P-513, IC-91729;	–	Negi and Muneem, 1998
		Barkha (RBU 38); Gujarat urd 1;	1	Goyal and Khan, 2010
	Resistant	Mash 391; IPU 11-02, Tripura Maskalai	1	Sandhu et al., 2012
Leaf crinkle virus	Tolerant	Mash 391	–	Sandhu et al., 2012
		KU-321, KU-1408, and KU-1375	1	Abhishek et al., 2020
	Resistant	IPU-96-6, IC-16511, NO-5131		Guljar et al., 2019
		MDU 1, Mash 479	1	Sandhu et al., 2012
Root Rot	Resistant	IPU-96-6, IC-16511, NO-5131	1	Guljar et al., 2019
		LBG 611	1	Goyal and Khan, 2010
Anthracnose	Tolerant	Mash 479; Mash 391	–	Sandhu et al., 2012
Wilt	Resistant	LBG 611; LBG 22	1	Goyal and Khan, 2010
Bruchids	Moderately resistant	TU 68	3	Subramaniyan et al., 2021
		IC 8219, UH 82-5, SPS 143	3	Ponnusamy et al., 2014
	Resistant	Mash 59, VM 2011, and VM 2166	1	Gupta and Kumar, 2006
		VM 2011 and VM 2164		Fernandez and Talekar, 1990
Stem fly	Resistant	BLG0069-1, BLG0036-1, BLG0079-1	1	Neupane et al., 2021
Spotted Pod borer	Resistant	LBG 762, LBG 726, LBG 747, LBG 744, and LBG 745	1	Krishna et al., 2006
	Moderately resistant	DU-1, DBGV-05, PU-30, PU-40	3	Manjunath and Mallapur, 2019

Black gram germplasm identified for abiotic stress resistance is summarized in **Table 5**. Vamban 2 variety has been reported as tolerant to drought stress in different studies and hence could be used as a parent to develop new varieties and genetic populations to further investigate the mechanism of drought tolerance in black gram (Goyal and Khan, 2010; Solanki et al., 2011 and Singh et al., 2016b). Other genotypes such as PGRU 95016, COBG05, IPU 99209, IPU 941, and IPU 243 were also reported as drought tolerant (Gurumurthy et al., 2019). The black gram varieties ‘J.L’, ‘PDU-1’ (Dash and Shree, 2013), ‘VBG-07-001’, and ‘VBG-06-010’ (Partheeban, 2017) were found to perform well in high-temperature regimes. Gupta et al. (2021) studied a panel of 97 diverse black gram genotypes for yield under heat stress and non-stress conditions in the field and identified eight highly tolerant lines (‘UPU 85-86’, ‘IPU 94-2’, ‘IPU 98/36’, ‘NO-5731’, ‘PGRU 95014’, ‘PGRU 95016’, ‘PLU 1’, and ‘BGP 247’). The trait-specific genotypes for waterlogging tolerance (Rana et al., 2016), photo-insensitivity (Singh et al., 2016b; Dash and Shree, 2013), and thermo-insensitivity (Singh et al., 2016b; Partheeban, 2017) were reported in different studies and are listed in **Table 6**.

**Table 6:** Genetic resources for major abiotic stresses in Black gram.

Trait	Level of tolerance	Identified genotype	Reference
Drought	Tolerant	Vamban 2	Singh et al., 2016b; Goyal and Khan, 2010; Solanki et al., 2011
		PGRU 95016, COBG05, IPU 99209, IPU 941, and IPU 243	Gurumurthy et al., 2019
		VBN(Bg)4 and VBN(Bg)6	Pandiyan et al., 2017
Thermo-insensitive	Tolerant	PGRU 95016, IPU 99-89, IPU 94-1, IPU 99-79, BGP 247, Pant Urd 31; VBG-07-001, VBG-06-010; UPU 85-86’, ‘IPU 94-2’, ‘IPU 98/36’, ‘NO-5731’, ‘PGRU 95014’, ‘PGRU 95016’, ‘PLU 1’, ‘BGP 247	Singh et al., 2016b; Partheeban, 2017; Gupta et al., 2021
	Moderately tolerant	VBN-6, COBG-11-02	Partheeban, 2017
Photo-insensitive	Tolerant	PGRU 95016, IPU 99-89, IPU 94-1, IPU 99-79, BGP 247, Pant Urd 31, and Pant U-40; PDU 1	Singh et al., 2016b; Dash and Shree, 2013
Waterlogging	Tolerant	EC319031–33	Rana et al., 2016
		TC-81855, TC-81860, TC-91567, TC-91927, TC-100190, TC-100193, TC-100353, N-47, N-830, P-513	Negi and Muneem, 1998

Black gram germplasms that are useful for nutritional quality improvement are shown in **Table 7**. The genotypes with high protein content such as LBG17, Malabiri local, Boudha local, and G. Udayagiri local with >25% protein (Kole et al., 2002) and K1 and IPU 11-02 with >24% protein content were identified in black gram (Rana et al., 2016). The genotypes UG-218 and HIM Mash were reported with better protein quality, especially high methionine and lysine content (Modgil et al., 2019). Accessions rich in iron (Fe) and zinc (Zn) were also reported in black gram, which could be used to develop biofortified varieties to achieve nutritional security by combating mineral malnutrition (Singh et al., 2017).

**Table 7:** Genetic resources for nutritional quality traits in black gram.

Trait	Quantification	Identified genotype	Reference
Protein	% Protein content	K1 (24.2%)	Rana et al., 2016
		IPU 11-02 (26.42%)	–
		LBG17 (28.4%), Malabiri local (26.3%), Boudha local (25.8%), and G. Udayagiri local (25.8%)	Kole et al., 2002
		Mash 479 (25.25%)	Sandhu et al., 2012
Methionine content	(g/100 g protein)	UG-218(0.54), HIM Mash (0.18)	Modgil et al., 2019
Lysine content	(g/100 g of protein)	UG-218(1.06), HIM Mash (1.57)	Modgil et al., 2019
Fe content	Fe (ppm)	COBG-653 (92.2), IPU-2-43(86.3), SHEKHAR2 (100.20), Mash-114 (97.9), PU-31 (97.54), PDU-1 (94.79), SPS 32(89.7), IPU 94-11 (90.3)	Singh et al., 2017
Zn content	Zn (ppm)	IPU-99-200 (47.5), SHEKHAR2 (60.5), YAKUBPUR2 (56.05), IPU-94-2 (44.48), PU-31 (50.25), PDU-1 (57.94), SPS 32(48.75), IPU 96-7(47.03)	Singh et al., 2017
Low oligosaccharides content	mg/gm	TU43-1 (26.64)	Souframanien et al., 2014

### 4.4 Popular varieties

In India, the establishment of the All India Coordinated Pulses Improvement Project (AICPIP) in 1967 provided breeders access to improved germplasm and an opportunity to test their improved breeding lines in multi-location trials across the country. The collective efforts of State Agriculture Universities and the Indian Council of Agricultural Research-Indian Institute of Pulses Research (ICAR-IIPR) resulted in the development and release of over 85 black gram varieties. Before the establishment of AICPIP, the early efforts of varietal development resulted in the release of popular varieties such as T 9, T 27, and T 77 (Gupta et al., 2001). T 9, a selection from the material collected from Bareilly (UP) in the early 1950s, has been used extensively by different breeding programs (Goyal and Khan, 2010). The varieties T 9, ADT 1, and Co 1 had made significant contributions to black gram production in India and were extensively used in the breeding program as parents to develop new varieties (Gupta et al., 2001). In Myanmar, six varieties were developed and released to farmers including Yezin-2, Yenzin-3, Yenzin-5, Yezin 6, Yezin-7, and Pale Tun. The first four varieties were selected from accessions P-45-1 (India), P-11-30 (Myanmar), P-69-354 (Myanmar), and LBG-17 (India). Yezin-7 was developed from hybridization and selection of the cross Yezin-4 and Yezin-6. Pale Tun is a mutant of P-11-30. In Thailand, farmers, traders, and users prefer varieties with large seed sizes. U-Thong 2 and Phitsanulok 2, pure-line varieties selected from Indian varieties 68/71 and BC48 (PI 288603) and released in the late 1970s and early 1990s, respectively, have been grown widely by the farmers for nearly 25 years due to high and stable yield and large seed size, compared to local varieties. At present, Chai Nat 80, a progeny derived from hybridization between a local variety (Prajeen) and an Indian variety (NBG 5), is the most popular cultivar in Thailand. In Australia, Regur released in the mid-1970s has been grown as a single black gram variety for approximately 35 years. The list of varieties developed in India, Bangladesh, Pakistan, Thailand, Australia, and Myanmar is presented in **Table 8**.

**Table 8:** List of varieties popularly grown in different places and different seasons.

Country	State	Varieties
India	Andhra Pradesh	L 35-5, T 9, Pant U 30, LBG 17 (Rabi), LBG 20, PS 1. LBG 623, LBG 402
	Assam	T 122, T 27, T 9, Pant U 19
	Bihar	Naveen, BR 68, Pant U 19, T 9, PS 1, PDU 1, PS 1
	Delhi	UG 218, T 9, PDU 1 (Spring)
	Gujarat	A 46-5, Zandewal, T 9, Pant U 30, G 75, PDU 1 (Spring)
	Haryana	Mash 1-1, Mash 48, T 9, UG 218, PDU 1 (Spring), Pant U 19
	Himachal Pradesh	Kulu 4, HPU 6, T 9
	Jammu & Kashmir	T 9, Maxh 1-1, Pant U 19
	Karnataka	Khargone 3, T 9, LBG 17 (Rabi)
	Madhya Pradesh	Gwalior 2, Khargone 3, No.55, T 9, Mash 48, Pant U 30, PS 1, PDU 1 (Spring), JU 2, JU 3
	Maharashtra	Sindhhera 1-1, No.55, D 6-7, T 9, Pant U 30, TAU 1, PDU 1 (Spring)
	Odisha	T 9, T 65, Pant U 30, Sarla
	Punjab	UG 218, T 9, Mash 1-1, Mash 48, PS 1, PDU 1 (Spring)
	Rajasthan	Krishna, Pant U 19, PDU 1 (Spring)
	Tami Nadu	ADT 1, CO 1, CO 2, CO 3, CO 4, CO 5, KM 1 KM 2, ADT 2, ADT 3, TMV 1, Pant U 30 LBG 17 (Rabi) CO 4, ADT 2, ADT 3, ADT 4, ADT 5, TMV 1 (Rice fallows)
	Uttar Pradesh	Type 9, T 27, T 65, Pant U 19, UG 218, Pant U 30, PS 1, PDU 1
	West Bengal	B 76, T 9, Pant U 19
Pakistan	–	Mash 97, Mash 2, Mash 3, CHAKWAL Mash, Mash 88
Bangladesh	–	BARI Mash-1 (*Panth*), BARI Mash-2 (*Saroth*), BARI Mash-3 (*Hemanta*) and BARI Mash-4, MAK-1, BINA Mash-1 (Mutant), MK-56, MK-61 &MK-83
Thailand	–	U-Thong 2, Phitsanulok 2, Chai Nat 2, Chai Nat 4, Chai Nat 80
Australia		Regur, Onyx-AU
Myanmar	–	Yezin-1, Yezin-2, Yezin-3, Yezin-4, Yezin-5, Yezin-6, Yezin-7, Pale tun, YB 9401-2-17

In India, over 60% of black gram varieties share type 9 (T9) as one of the ancestors in their pedigree (Singh et al., 2016a). The pedigree analysis of advanced breeding lines entered for the Initial Varietal Trial in AICPIP from 1996 to 2005 revealed that T9 was used as one of the parents in over 78% of the advanced breeding lines (Katiyar et al., 2008). Another variety, D 6-7, was used as a parent in more than 12% of advanced breeding lines. The use of the limited number of parents by the breeding programs could be one of the reasons behind the narrow genetic base of available varieties. Therefore, it is an urgent need for the breeding programs to focus more on the use of diverse genetic material and recycling the newly developed advanced breeding lines into crossing nurseries.

## 5 Genomics

### 5.1 Genomic resources

Genetic improvement in several food legumes by either traditional or molecular approaches has been hindered due to the absence of genomic resources till the last decade (Varshney et al., 2009). The DNA marker techniques have been developed in various crops including food legumes owing to the recent developments in molecular biology. It includes the development of several different generations of molecular markers, molecular characterization of germplasm, and the development of genetic and quantitative trait locus (QTL) maps along with trait-linked diagnostic markers (Boukar et al., 2019). Genetic linkage maps and diversity within germplasm collections in black gram have been studied primarily using random amplified polymorphic DNA (RAPD) (Souframanien and Gopalakrishna, 2004), inter simple sequence repeat (ISSR) (Souframanien and Gopalakrishna, 2004), amplified fragment length polymorphism (AFLP), and SSR markers (Chaitieng et al., 2006).

Several efforts have been made to develop genomic resources and use them to assess the diversity at the genomic level in black gram. The DNA polymorphism in 18 elite genotypes using 25 RAPD and 16 ISSR markers reported 44 out of 104 scoreable fragments for RAPD and 55 out of 101 bands for ISSR as polymorphic (Souframanien and Gopalakrishna, 2004). ISSR markers have been effectively used for repeat motif analysis as well as varietal identification in black gram (Ranade and Gopalakrishna, 2001). The genetic diversity of 26 black gram landraces using AFLP markers revealed 74.5% to 93% polymorphism (Sivaprakash et al., 2004).

Since there were not many polymorphic markers in black gram, the transferability of SSR markers from other *Vigna* species to a black gram was tested and reported several polymorphic markers. Chaitieng et al. (2006) tested the amplification and polymorphism of 211 genomic SSR markers from azuki beans in cultivated and wild black and found that 73% and 50% of the markers were amplifiable and polymorphic, respectively. Chankaew et al. (2014) analyzed the application of 1,429 azuki bean genic SSR markers in the same black gram accessions used by Chaitieng et al. (2006) and found that 84% of the markers were amplifiable but that only 13% of them were polymorphic. Tangphatsornruang et al. (2009) detected amplification and polymorphism of 127 mungbean genomic SSR markers in cultivated and wild black gram and found that 66% and 50% of the markers were amplifiable and polymorphic, respectively. In a similar study, Somta et al. (2009) reported that as high as 92% of 85 genic SSR markers from mungbean tested in the same black gram materials reported by Tangphatsornruang et al. (2009) were amplifiable and 34% were polymorphic. Sixty-five SSR markers of cowpea were tested across different *Vigna* species and reported amplification of 85% (55) markers in black gram (Gupta and Gopalakrishna, 2010). The molecular diversity of six MYMD-resistant and MYMD-susceptible genotypes each from mungbean and black gram using 24 cowpea resistance gene analog (RGA) markers grouped two resistant (IPU 02-33 and IPU 6-02) and two susceptible (LBG 20 and T9) black gram genotypes into distinct clusters (Narasimhan et al., 2010). However, the limited number of SSR markers in these legumes hindered the genomic studies in the black gram.

The emergence of advanced sequencing technologies including next-generation sequencing (NGS) and third-generation sequencing (TSG) in the 2000s has revolutionized genome study in living organisms. NGS is able to sequence millions of short fragments of DNA in parallel. It provides a rapid, inexpensive, and comprehensive analysis of the genomes of individual organisms as well as complex populations. NGS is also a powerful tool in the discovery and genotyping of large numbers of single-nucleotide polymorphisms (SNPs) at a drastically reduced expense (Elshire et al., 2011) and in the construction of whole-genome sequencing, thus enhancing the genetic gain in breeding. Third-generation sequencing (TGS) (also known as single-molecule sequencing) sequences large numbers of long fragments of DNA, which is highly useful for producing high-quality genome assemblies providing scientists to explore genomes at an unprecedented resolution. The transcriptome sequencing of immature seed tissues of TU94-2 through Illumina paired-end sequencing technology generated 17.2 million paired-end reads and 48,291 transcript contigs (TCS) that could be useful for gene discovery and genic-SSR markers (Souframanien and Reddy, 2015). Another study on the transcriptome sequence of immature seed tissue in wild black gram developed a large number of genic SSR and SNP markers (Raizada and Souframanien, 2019). Genetic relatedness among 27 black gram genotypes was also evaluated using 19 genic SNPs through high-resolution melt (HRM) analysis (Raizada and Souframanien, 2021).

A whole-genome sequence (WGS) or reference genome sequence of a species is an indispensable genomic resource for gene discovery. WGSs of black grams have been developed recently. Pootakham et al. (2020) produced a high-quality, chromosome-level assembly of 499 Mb comprising 11 pseudomolecules from the black gram variety Chai Nat 80 (CN80) using NGS and TGS technologies. The genome annotation contained 32,729 predicted gene models, of which 29,411 (89.86%) were protein-coding genes. Shortly, Souframanien et al. (2021) constructed a draft genome sequence of 502 Mb of black gram variety Pant U-31 with 42,115 predicted genes. Ambreen et al. (2022) constructed a draft reference-guided genome assembly of black gram genotype ‘Uttara’ (IPU 94-1; known for its high resistance to Mungbean Yellow Mosaic Disease), with a cumulative size of 454 Mb and 28,881 predicted genes, of which 444 Mb was anchored on 11 chromosomes. Naito et al. (2022) constructed a draft genome sequence of 475 Mb of black gram variety Subsmotod with 28,227 predicted genes. The size of these black gram genome assemblies is only 79%–87% of the estimated genome size of the black gram (574 Mb; Arumuganathan and Earle, 1991). Therefore, a large part of the black gram genome still cannot be captured. In addition, the number of predicted genes in these genomes is highly different, albeit this may reflect the high genome variations among the black gram varieties. Nonetheless, these WGSs are useful genomic resources to hasten the understanding of evolutionary relationships, the development of diagnostic molecular markers, and the identification of novel genes and haplotypes in black gram.

### 5.2 Quantitative trait locus mapping and trait-specific markers

The development of genomic resources opens opportunities for the construction of linkage maps and the identification of quantitative trait loci and diagnostic molecular markers for desirable traits (He et al., 2014). High-quality genome assembly together with information about genomic variations from germplasm enabled the use of novel breeding tools such as genomic selection (GS), marker-assisted selection (MAS), marker-assisted recurrent selection (MARS), and marker-assisted backcrossing (MABC) (Ribaut et al., 2010) and further comparative genomics and phylogenetic research into legumes (Pandey et al., 2016). Due to limited genomic resources for the black gram, especially polymorphic DNA markers, in the past, there have been few reports on gene and QTL mapping in this crop. A genetic linkage map is a basic tool for gene and QTL mapping. The first genetic linkage map of the black gram was reported in 2006 when Chaitieng and her colleagues developed linkage maps for the black gram using only 148 marker loci including 59 RFLP, 61 SSR, 27 AFLP, and one morphological marker (Chaitieng et al., 2006). Two years later, another black gram linkage map was developed using 428 marker loci (254 AFLP, 86 RAPD, 41 ISSR, and 47 SSR markers), but unfortunately, most of them were dominant (Gupta et al., 2008). Recently, a high-density linkage map of 3,675 SNP markers from specific-locus amplified fragment sequencing was developed (Somta et al., 2019). All of these maps were constructed based on a population derived from crossing between cultivated and wild black gram.

Most of the gene and QTL mapping studies in black gram involved the resistance to MYMD and bruchid (*C. maculatus*). By using bulked-segregant analysis (BSA), Gupta et al. (2013b) identified SSR marker CEDG180 linked to MYMD resistance. ISSR marker ISSR8111357 was identified to be linked to the MYMD resistance gene with a 6.8-cM distance, and the marker was converted into sequence characterized amplified region (SCAR) marker for validation on other MYMD resistant and susceptible genotypes (Souframanien and Gopalakrishna, 2006). Through BSA using an F_2_ population of a cross between T9 (MYMD resistant) and LBG-759 (MYMD susceptible), SSR marker VR9 was found to be linked to MYMD resistance (Naik et al., 2017). In another study, SSR marker CEDG185 was identified as linked with the MYMD resistance by BSA (Rambabu et al., 2018). Two major QTLs, *qmymv2_60* and *qmymv10_60*, governing resistance to MYMD disease were mapped onto LG2 and LG10 with 20.90% and 24.90% phenotypic variation explained (PVE), respectively (Vadivel et al., 2021). Validation of these QTLs in two other mapping populations confirmed the presence of *qmymv10_60*. Similarly, a study on the F_2_ population derived from a cross between MDU 1 (susceptible) and TU 68 (resistant) identified one major QTL *qMYMVD_60* on LG10 responsible for 21% PVE of MYMD variation. This QTL was delimited by SSR markers CEDG180 and CEDG116 (Subramaniyan et al., 2022). Since the QTL *qmymv10_60* was confirmed and validated for its association with MYMD resistance in different studies, this QTL can be one of the potential regions for fine mapping and identification of diagnostic markers and haplotypes for MAS and haplotype-based breeding approaches.

By utilizing the genetic map developed by Gupta et al. (2008) (see above), QTLs *Cmrae1.1* and *Cmrae1.2* on LG3 and LG4, respectively, were detected for the percent adult emergence, and six QTLs (two (*Cmrdp1.1* and *Cmrdp1.2*) on LG1, three (*Cmrdp1.3*, *Cmrdp1.4*, and *Cmrdp1.5*) on LG2, and one (*Cmrdp1.6*) on LG10) were identified for developmental period with PVE ranging from 8.4% to 16.4% (Souframanien et al., 2010). By using the high-density SNP-based linkage map, two QTLs, *qVmunBr6.1* and *qVmunBr6.2*, located approximately 10 cM apart on LG6 were identified for a percentage of damaged seeds and infestation severity progress (Somta et al., 2019). Comparative genome analysis revealed *qVmunBr6.1* and *qVmunBr6.2* as new loci for *C. maculatus* resistance in *Vigna* species (Somta et al., 2019).

QTLs for seed weight were detected in black gram using *mog* (*multiple-organ gigantism*) mutant as the large-seeded parent. The *mog* mutant expressed extra-large size of seeds, leaves, and stems. QTL mapping and gene transformation studies revealed that *mog* gene accounted for approximately 66% of seed weight variation in the mapping population and additive gene effect of the *mog* increased 2.0 g of seed weight and that *mog* is a *VmPEAPOD* (*VmPPD*) gene (Naito et al., 2017). Loss of function caused by an 8-bp deletion on the *VmPPD* resulted in the *mog* phenotype (Naito et al., 2017). Later, major QTLs for the multiple-organ gigantism (MOG) phenotypes were reported on LG6 with the pleiotropic effect on different traits ranging from 15% for plant height to 40% for leaf size (Somta et al., 2020). The QTL for the MOG phenotype was also reported to contribute approximately 30% PVE for seed weight (Somta et al., 2020). In another study, QTLs for seed weight were identified using two F_2_ populations derived from crossing between cultivated and wild black grams in which 10 QTLs on seven linkage groups (LGs) were detected in total for the seed weight (Lomlek et al., 2023). The QTLs explained between 5.07% (*qSd100wt7.1+*) and 34.20% (*qSd100wt10.2+*) of the weight variation, depending on the population. Genes encoding for pentatricopeptide repeat (RPP)-containing protein, *WRKY46*, glutathione *S*-transferase U9, NAC domain-containing protein 100, calcineurin B-like protein (CBL)-interacting protein kinase, cyclin-D6-1, kinesin, histidine phosphotransfer protein, AUXIN RESPONSE FACTOR 2A, and WAVE-DAMPENED2 (WVD2)-like 4 protein are candidate genes for seed weight QTLs (Lomlek et al., 2023). Two minor QTLs for pod dehiscence, *qPdt3.1−* and *qPdt5.1−*, on LGs 3 and 5, respectively, with ≤10% PVE and a single QTL with large effect controlling approximately 20% PVE for seed dormancy were also reported in black gram (Somta et al., 2020). The QTLs detected for pod dehiscence in black gram were different than those reported in other closely related *Vigna* species including mungbean (Isemura et al., 2012), adzuki bean (Kaga et al., 2008), moth bean (Yundaeng et al., 2019), and cowpea (Kongjaimun et al., 2012). Recently, QTL analyses using dense SNP-based linkage maps of two mapping populations identified 12 different QTLs with PVE of 3.4% to 38.0% for flowering time in the black gram (Suamuang et al., 2023). Genes encoding zinc finger protein 10, F-box protein SKIP14, and NAC domain-containing protein 37-like are candidate genes at major QTLs for the flowering time (Suamuang et al., 2023). None of the candidates are the same as those for flowering time in other *Vigna* species. In addition, using the same populations and linkage maps as that reported by Suamuang et al. (2023), 10 QTLs were detected for seed weight, and three were detected for seed dormancy. Depending on populations, QTLs explained between 5.07% (*qSd100wt7.1+*) to 34.20% (*qSd100wt10.2+*) of the seed weight variation and 10.18% (*qSdwa6.1−*) to 43.81% (*qSd100wt6.2−*) of the seed dormancy variation (Lomlek et al., 2023). Several candidate genes were identified including *WVD2-LIKE 4* for seed weight that appeared to be an ortholog with a candidate gene for the seed weight in mungbean.

## 6 Breeding

Considering the low rate of genetic gains and on-farm yield levels, higher seed yield with early maturity (65–75 days), photo–thermo insensitivity, rapid plant growth, high biomass, high harvest index, increased seed size, synchronous maturity, resistance to MYMD, ULCD, PM, and CLS, and tolerance to drought, heat, and salinity stresses are some of the major breeding objectives for black gram breeding programs across the world. In addition to these, high protein content, higher amounts of sulfur-containing amino acids, and high Fe and Zn contents are some of the major nutritional quality traits focused on by the breeding programs. The varieties with erect semi-determinate plant types are becoming popular due to their suitability for cultivation in the sole cropping system and mechanical harvesting.

Intensive breeding programs of black gram are in India. Breeding methods such as introduction, mass selection, pure line selection, pedigree selection, backcrossing, and mutation breeding are widely used by most of the breeding programs in India and elsewhere. Initially, the varieties were developed through direct selections from local landraces and introduced germplasms. Over 50% of varieties in India from 1949 to 2000 were developed through selections from local materials (Gupta et al., 2020). T9 is one of the popular varieties developed through the introduction and mass selection, whereas ADT 1, CO 1, CO 2, CO 3, CO 5, and ADT 3 were developed through pure line selection from local populations (Gupta and Kumar, 2006; Tickoo et al., 2006). Later, the emphasis was on the use of hybridization-based techniques to create variability and to combine multiple traits in a single variety to cater to the needs of different production environments (Gupta et al., 2020). In India, KM 1 was the first variety developed through hybridization in 1977. VBN 6, 7, 8, 9, 10, and 11 are some of the varieties recently developed using hybridization followed by selection (Geetha et al., 2020). The hybridization-based approaches require prior accurate information about parental genotypes, genetics of the trait to design a sound breeding strategy, precise phenotyping procedures, and facilities for testing of segregating and advanced breeding material. These approaches have contributed to generating new recombinants with desirable traits and to the development of several popular varieties across different countries (Dikshit et al., 2020). Most of the varieties developed in black gram during the last two decades are based on recombining the traits through hybridization and phenotypic selections in early and advanced generations. The multiparent-based hybridization is now being explored by the black gram breeding programs to develop varieties with a broader genetic base. In Thailand, black gram breeding began in the mid-1970s with the major aims of improving plant type, earliness of flowering and maturity, and seed size and yield. At the early stages of breeding, germplasm from the World Vegetable Center was introduced and selected by mass selection and pure line selection leading to the release of two varieties, U-Thong 2 and Phitsanulok 2, which have been utilized for export for approximately 30 years. All the recent varieties were developed by hybridization and selection using exotic and local germplasm. At the World Vegetable Center, black gram lines (AVUB 2001 to AVUB 2030) were recently developed with improved protein quality, and resistance to bruchids and MYMD, from crosses involving VM 2164 with Mash1-1 (Boddepalli et al., 2021).

Considering the narrow genetic base of cultivated black gram and cross incompatibility with many of the wild *Vigna* species, mutation breeding using various mutagens has been adopted in black gram to create variability and develop improved varieties. It has played a remarkable role in genetics, genomics, and breeding research across different crops (Kharkwal and Shu, 2009). Globally, mutation breeding has contributed to the commercial release of 3,348 plant varieties across 229 different plant species in more than 75 countries (FAO/IAEA Mutant Variety Database, 2020). Approximately 18 varieties have been released through mutation breeding in black gram, and all the varieties except one have been released from India. The gamma rays were used effectively in black gram to create variability for chlorophyll content (Usharani and Kumar, 2015; Ramchander et al., 2017), methionine content (Arulbalachandran et al., 2009), pods per plant, number of seeds per plant, number of primary branches, pod length (Usharani and Kumar, 2015; Anitha and Mullainathan, 2018), seed size (Chinchest and Nakeeraks, 1991), and seed yield (Raina et al., 2018). The gamma irradiation treatment successfully created inter-population that led to the selection of two novel mutants, i.e., G7 and G13 derived from ADT 3, and G34 from TU 37-9 with improved yield and stable performance across environments (Dasarathan et al., 2021). The nutritional and battering qualities of varieties MDU 1 and VBN (Bg) 4 were increased through electron beam and gamma-ray mutants. The mutant lines, *viz.*, ACM-014-021, ACM-015-015, ACM-15-023, ACM-015-013, ACM-015-003, ACM-015-030, ACM-014-006, and ACM-014-007, were identified to have superior albumin content, globulin content, total soluble protein, arabinose content, 100-seed weight, and seed yield per plant (Vanniarajan et al., 2021). Apart from the conventional electromagnetic radiations, like X-ray and gamma ray, the electron beam is now an alternative source of energy to induce mutations. In black gram, electron beam irradiation showed increased effectiveness and efficiency in the induction of chlorophyll and morphological mutants in comparison to gamma rays (Souframanien et al., 2016).

**Figure 1:**
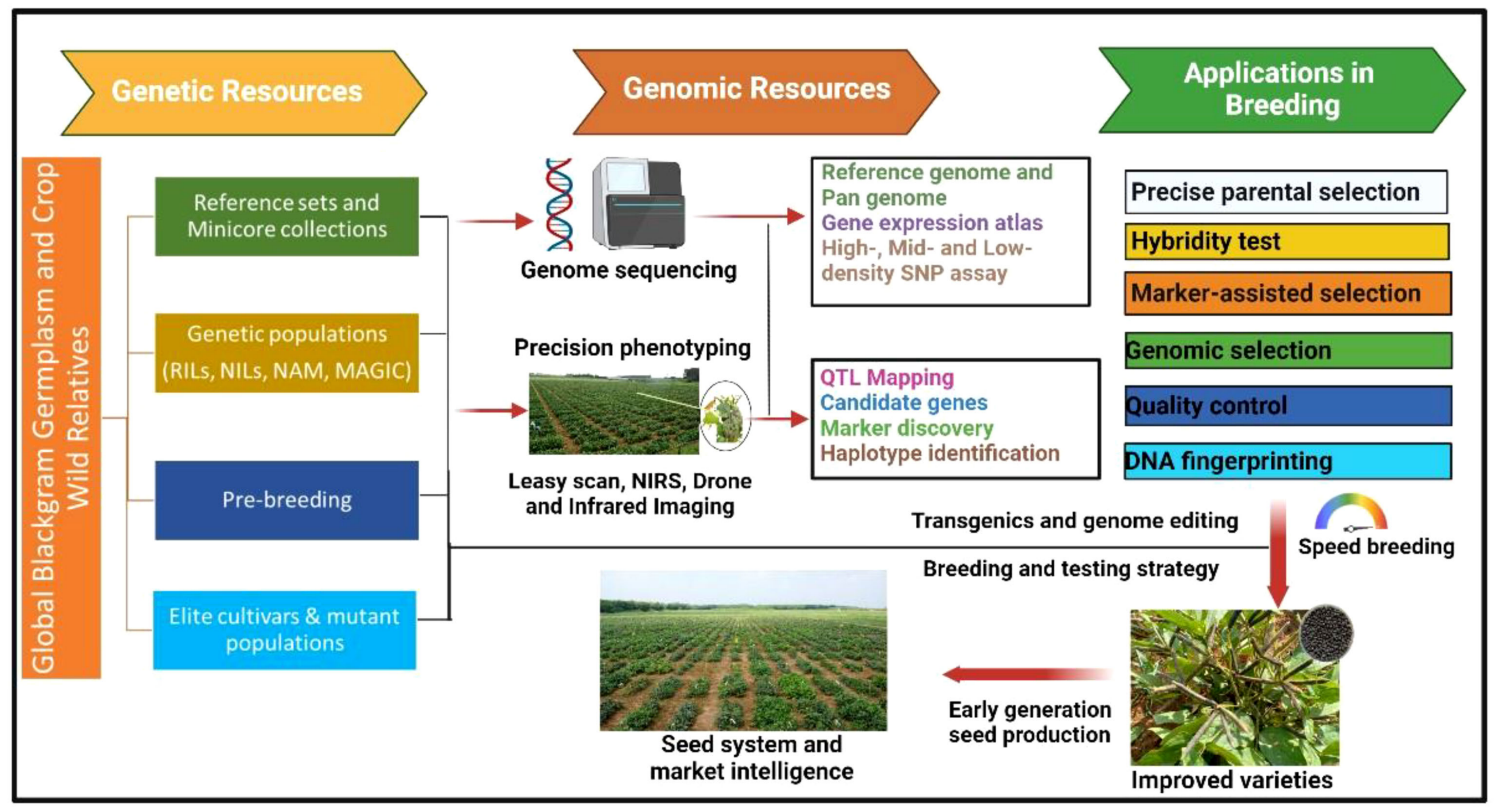
Genetic and genomic resources and their applications in black gram breeding for accelerating the genetic gains. RIL, recombinant Inbred Lines; NIL, near-isogenic lines; NAM, nested association mapping; MAGIC, multiparent advanced generation intercross).

Ethyl methanesulfonate (EMS) was also successfully used to create variability for number of seeds per pod, number of primary branches, plant height (Usharani and Kumar, 2015; Kuralarasan et al., 2018), protein content, nitrate reductase activity (Thilagavathi and Mullainathan, 2011), methionine content (Arulbalachandran et al., 2009), and seed yield (Verma et al., 2018). Some of the other mutagens such as sodium azide (Raina et al., 2018), diethyl sulfate (Anitha and Mullainathan, 2018), colchicine, and electron beam (Thilagavathi and Mullainathan, 2011; Veni et al., 2017) were also successfully used in black gram. In India, the Bhabha Atomic Research Centre (BARC) facilitates mutation breeding in several crops including black gram. The varieties developed through mutation breeding include CO 4, Vamban 2, TU94–2, TAU-1, TAU 2, TPU-4, Ujala (OBG17), and Prasad (B3–8-8) (Reddy and Dhanasekar, 2007). The variety CO 4 has early maturity (70–80 days) along with a large seed size (6 g/100 seeds) and is resistant to powdery mildew. Three varieties, namely, Sarla, Prasad, and Vamban 2, are the mutants of a popular variety T9; Sarla and Prasad have tolerance to MYMD, whereas Vamban 2 has tolerance to drought stress. Ujala (OBG17), a mutant variety developed from Prasad (B3–8-8), offers resistance to MYMD and CLS (Gupta et al., 2020). The breeding methods based on phenotypic selections were quite successfully used for those traits where phenotypic selection is precise and easy. The phenotyping for several desirable traits (such as biotic and abiotic stresses) and nutritional quality traits is tedious and time- and resource-consuming and largely depends upon the environmental conditions and phenotyping approaches. This is one of the reasons behind low genetics gains for such desirable traits in black gram and several other crops leading to stagnant yield levels across major growing countries. In contrast to this, the trait-linked molecular markers offer a precise and accurate selection of parents and individuals of segregating populations that can supplement the phenotypic selection for target traits. MAS is one of the useful and precise methods of selection of individuals with desirable traits for genetic improvement and is now being widely used in multiple legumes such as chickpea (Thudi et al., 2014), pigeonpea (Pazhamala et al., 2015), mungbean (Schafleitner, 2020), and groundnut (Varshney et al., 2014; Deshmukh et al., 2020). Although efforts were made in the past for the identification of molecular markers linked to the desirable traits in black gram, none of them could be successfully deployed for MAS in the breeding programs. The PCR-based markers, particularly SNPsv are widely preferred by the breeding programs, as they are cost-effective for genotyping in large segregating populations (Bohar et al., 2020). A flowchart of genetic and genomic resources and their applications in black gram breeding is summarized in **Figure 1**. The advancement in the identification of diagnostic markers and low-cost genotyping platforms for low-, mid-, and high-density genotyping assays and high-throughput phenotyping tools are expected to help breeding programs enhance genetic gains in black gram.

## 7 Transgenic research and scope of genome editing

The desirable genes from different species or genera have been successfully transferred into high-yielding cultivars in many crop species; however, most of them are not yet commercialized due to environmental biosafety and regulatory policies across many countries (Zhang et al., 2018). In black gram, the protocols for *in vitro* regeneration systems and genetic transformations by *Agrobacterium tumefaciens* have been already developed and used by Saini et al. (2003). The transgenic black gram was developed through the transformation of shoot apices with *A. tumefaciens* (strain EHA105) containing a binary vector pKSB (Saini and Jaiwal, 2005). The binary vector harbored bialaphos resistance (bar) and commonbean α-amylase inhibitor-1 (αAI-1) genes leading to increased transformation efficiency of up to 6.5%. The successful transformation with 7.6% frequency was achieved using a cotyledonary node and *A. tumefaciens* (LBA4404) carrying the binary vector pME 524 (with nptII, bar, and uidA genes). Attempts were also made to incorporate salt tolerance by transforming black gram with glyoxalase I gene derived from *Brassica juncea* and the constitutive CmYLCV promoter (Bhomkar et al., 2008). A plasmid pGJ42 harboring neomycin phosphotransferase (nptII) selectable marker gene, the barley antifungal gene chitinase (AAA56786), and ribosome-inactivating protein (RIP; AAA32951) were successfully used for the transformation of black gram to develop resistance against corynespora leaf spot disease caused by *Corynespora cassiicola* (Chopra and Saini, 2014). The protection from diseases among transformed lines varies from 27% to 47% compared to wild-type plants. One of the Alto Keto Reductase (AKR) genes, ALDRXV4 was extracted from *Xerophyta viscosa* and transferred to the black gram through *agrobacterium* mediation. The T1 individuals were resistant to drought, salt, and H_2_O_2_-induced oxidative stresses by reducing the accumulation of toxic metabolites and upregulating the sorbitol accumulation in the plants (Singh et al., 2016b). Genome-editing tools such as CRISPR/Cas9 are becoming popular functional genomics tools among researchers, as they can create novel genetic variations with the deletion of harmful or the addition of desirable traits in plants with precision and efficiency (Chen and Gao, 2014). It is a rapidly developing technique being used for different genetic manipulation, including generating knockouts, making precise modifications, creating multiplex genome engineering, or activating and repressing genes (Arora and Narula, 2017). The use of genome editing in various crops is increasing (Chen and Gao, 2014; Zhang et al., 2018); however, there has been limited success in legume species. The CRISPR/Cas9-mediated genome editing has been successfully reported in *Medicago truncatula* (Michno et al., 2015; Confalonieri et al., 2021), soybean (Sun et al., 2015; Han et al., 2019; Bao et al., 2020), red clover (Dinkins et al., 2021), cowpea (Ji et al., 2019; Che et al., 2021), and chickpea (Badhan et al., 2021). In cowpea, the symbiosis receptor-like kinase target gene VuSYMRK edited gene that controls nodule symbiosis exhibited complete inhibition in nodule formation (Ji et al., 2019). This technology in association with base editors and prime editing could be used for *de novo* domestication of crop wild relatives (CWRs) of underutilized legumes and “reengineering of metabolism” to increase resilience and enhance nutritive value (Gasparini et al., 2021; Nasti and Voytas, 2021). The genome-editing technologies could significantly contribute to legume improvement including black gram to enhance productivity through improving biotic and abiotic stress tolerances (Jha et al., 2022; Mahto et al., 2022; Singh et al., 2023).

## 8 Future prospects

Legumes are versatile crops that contribute to mitigating the global food security challenges under changing climate scenarios. The development of genomic resources in legumes including black gram could help breeders to use genomic-assisted breeding tools such as diagnostic molecular markers for target traits to make precise selections in early generations. The developments in the genome sequence could enable researchers to develop low-, mid-, and high-density SNP assays that can be used for new genomic discoveries and deployment of genomic predictions for selection decisions based on breeding values. These genomic-based predictions and speed breeding approaches could enhance genetic gain in legumes (Jha et al., 2022). The novel insights into genomic variations for target traits, their evolution, domestication events, and diversification could be generated through genomic sequencing of CWRs, landraces, and improved breeding lines. Efforts are needed for the development and integration of high-throughput precision phenotyping tools to accelerate the genetic gains for complex traits. A deeper understanding of the mechanism of resistance/tolerance to various biotic and abiotic stresses will be useful in designing a sound breeding strategy. In addition to genetic gains for yields, the improvement in nutritional quality traits such as protein content and quality, and high iron and zinc content could be traits of interest to achieve nutritional security, especially in developing countries. The greater resilience of black gram to changing climatic conditions offers an opportunity for horizontal expansion of the area to enhance environmental sustainability, particularly in cereal-based cropping systems.

## Author contributions

RN: Conceptualization, Writing – original draft, Writing – review & editing, Funding acquisition. SC: Writing – original draft, Writing – review & editing. ND: Writing – original draft. AS: Writing – original draft. AG: Writing – original draft. NB: Writing – original draft. HP: Writing – original draft. RS: Writing – original draft, Writing – review & editing. SJ: Writing – original draft, Writing – review & editing. PS: Writing – review & editing.
